# Impact of Game-Inspired Infographics on User Engagement and Information Processing in an eHealth Program

**DOI:** 10.2196/jmir.5976

**Published:** 2016-09-22

**Authors:** Maria Leonora G Comello, Xiaokun Qian, Allison M Deal, Kurt M Ribisl, Laura A Linnan, Deborah F Tate

**Affiliations:** ^1^ School of Media and Journalism University of North Carolina at Chapel Hill Chapel Hill, NC United States; ^2^ Lineberger Comprehensive Cancer Center University of North Carolina at Chapel Hill Chapel Hill, NC United States; ^3^ Gillings School of Global Public Health University of North Carolina at Chapel Hill Chapel Hill, NC United States

**Keywords:** infographics, game design, eHealth, personalized feedback, visuals

## Abstract

**Background:**

Online interventions providing individual health behavior assessment should deliver feedback in a way that is both understandable and engaging. This study focused on the potential for infographics inspired by the aesthetics of game design to contribute to these goals.

**Objective:**

We conducted formative research to test game-inspired infographics against more traditional displays (eg, text-only, column chart) for conveying a behavioral goal and an individual’s behavior relative to the goal. We explored the extent to which the display type would influence levels of engagement and information processing.

**Methods:**

Between-participants experiments compared game-inspired infographics with traditional formats in terms of outcomes related to information processing (eg, comprehension, cognitive load) and engagement (eg, attitudes toward the information, emotional tone). We randomly assigned participants (N=1162) to an experiment in 1 of 6 modules (tobacco use, alcohol use, vegetable consumption, fruit consumption, physical activity, and weight management).

**Results:**

In the tobacco module, a game-inspired format (scorecard) was compared with text-only; there were no differences in attitudes and emotional tone, but the scorecard outperformed text-only on comprehension (*P*=.004) and decreased cognitive load (*P*=.006). For the other behaviors, we tested 2 game-inspired formats (scorecard, progress bar) and a traditional column chart; there were no differences in comprehension, but the progress bar outperformed the other formats on attitudes and emotional tone (*P*<.001 for all contrasts).

**Conclusions:**

Across modules, a game-inspired infographic showed potential to outperform a traditional format for some study outcomes while not underperforming on other outcomes. Overall, findings support the use of game-inspired infographics in behavioral assessment feedback to enhance comprehension and engagement, which may lead to greater behavior change.

## Introduction

Online health behavior assessments have become important tools for monitoring and motivating individual health behavior change [1-3]. Behavior change is possible when assessments provide a combination of information and personalized feedback [4-6]. However, to be effective, the feedback must be clear and engaging. Users who find the information confusing, uninteresting, or even discouraging may be deterred from further interaction. Thus, in addition to being comprehensible, content should also be engaging to retain as many users as possible who may benefit from continued participation [7].

This study focused on the potential of infographics to address these communication challenges when used to deliver feedback following online health behavior assessments. Infographics have been defined as a visualization of data or ideas to convey information in a way that is easily understood [8]. Although infographics are frequently used to deliver health information, there is a lack of research on the conditions under which different formats may be most effective [9]. We addressed this gap by examining multiple infographic formats as part of feedback for 6 health behaviors. The study included formats inspired by gamification, which is the use of game design elements in nongame contexts [10]. The key issue was whether game-inspired infographics (ie, infographics inspired by aesthetic and other elements of games) would be more effective at facilitating comprehension, positive attitudes toward the information, positive emotional responses, and other outcomes than the more traditional formats that have been used to represent behavioral feedback.

We explored this issue through pretesting visual elements for use in the Carolina Health Assessment and Resource Tool (CHART). CHART is an online tool developed at the University of North Carolina at Chapel Hill, USA, that offers evidence-based assessment for health behaviors that have been linked to the leading causes of death due to chronic disease in the United States [11]. The 6 behavioral modules addressed in this study were tobacco use, alcohol use, physical activity, vegetable consumption, fruit consumption, and weight management. CHART provides feedback that includes a statement of the individual’s level of behavior (based on questions answered in the assessment) relative to the recommended level for that behavior. Although CHART output includes other personalized feedback components, this study focused only on the display of information regarding behavior relative to the recommended level.

We conducted formative research to guide selection of simple visuals that would accompany this feedback. Formative research refers to the stage prior to implementation of a health communication effort in which messages and strategies are evaluated for the likelihood of achieving intended effects [12]. Strategies can include experimental assessment of stimuli (sometimes called pretesting) to identify promising formats [13]. In particular, the evaluation of visual elements in the formative research stage is advocated by the US National Cancer Institute [14] for effective health communication; however, the process and results of such pretests are not often reported. Thus, this study contributes to knowledge of best practices in the development and systematic assessment of health infographics for use in online environments.

### Visuals and Infographics in Health Communication

Infographics are an increasingly common feature in the online media landscape because of their reputation for presenting information in a visually compelling and easy to process manner [8]. The most recent reviews of the use of infographics and other visuals in health contexts [15-17] suggest that visuals add to the impact of health information in terms of attention, comprehension, recall, intention, and behavior change. For people with low health literacy, the beneficial effect of visuals as an aid to comprehension is even more pronounced [17]. Further, certain kinds of visuals such as avatars in risk infographics may help people better comprehend how statistical risk information can apply to an individual [18]. It should be noted that many of these studies involved the delivery of quantitative risk information (eg, number of people affected by a type of cancer in a population) and not information on levels of individual behavior relative to a recommended level (*personalized* behavioral feedback), which was the focus of our study. Given the importance of tailoring in eHealth interventions [19], our study makes an independent contribution by examining infographic effects in the context of promoting individual risk factor change.

At the most basic level, studies have shown that adding visuals to text results in desirable outcomes from a health promotion perspective. For example, patients receiving discharge information as text with visuals had better outcomes (compared with those receiving text-only) in terms of attention to information, recall of information, and adherence to instructions [20]. An explanation for the superiority of formats that combine visuals with text over text-only is based on dual coding theory [21], which proposes that there are 2 cognitive systems, one specialized for the processing of language and the other for nonlanguage-based information. Although the systems are distinct, activity in one system can initiate activity in the other. Thus, information combining text with visuals is more likely to be encoded and comprehended than is information presented in text only [22].

Graphical formats may also influence perceptions of risks in several ways. For example, some formats can make some aspects of risk information more salient than others. A systematic review [15] showed that graphs that visualize part-to-whole relationships may draw attention to the relationship between the number of people affected by a hazard and the entire population at risk, whereas graphs that show only the number of people at risk may increase perceived risk and increase the likelihood of protective behavior. Thus, visuals have a role in framing risk information by providing cues to interpretation that may not otherwise be present. Also, the visual complexity of displays can influence perceptions of risk. Displays of quantitative risk information that contained animation resulted in worse performance (compared with a static display) on indicators of comprehension [23]. Thus, although more complex displays might seem to be preferable given a higher potential of being encoded (as would be suggested by dual coding theory), in some cases, simpler displays may be optimal.

Although assessments of graphs tend to focus on accuracy of perceptions and other cognitive outcomes, it is also important to look at the appeal of visual elements. In a real-world setting, people simply may not accept or attend to graphics they do not like [15]. This may be the case in self-guided assessments where people can exit at any time. Ancker and colleagues [15] found that participants preferred simple graphics and human figures over nonhuman figures. For example, in a qualitative study evaluating the responses of women to visual formats used to convey breast cancer risk, human figures were preferred over bar graphs because the human figures were perceived as relatable and as conveying a meaningful message [24].

A theoretical model of persuasion that provides a framework for thinking about both cognitive and affective outcomes as part of a larger process of persuasion is the hierarchy of effects model [25], which conceptualizes comprehension as one step among many in the behavior change process. Given the roots of the model in consumer behavior research, a key construct within the model is attitude toward the advertisement, an affective construct representing feelings of favorability that can be used in the assessment of advertising and other materials designed to have persuasive effects (eg, health messages). Studies have shown that attitude toward the advertisement mediates the effects of exposure on brand perceptions and purchase intentions [26-29] and on actual behavior [29]. In a health framework, positive evaluations of advertisements have an impact on perceptions of health-relevant product categories [30]. Although infographics are not commonly associated with advertising or promotion, the personalized feedback accompanying health behavior assessments is intended to promote progress toward healthy behavioral outcomes. Therefore, infographics used in such feedback should be evaluated for their impact on attitudes and emotions so that their role in supporting behavior change can be better understood.

### Game-Inspired Design

The infographics employed in this study were inspired by concepts underlying gamification, which has been defined as the use of game features in nongame contexts to motivate and engage users [10]. Organizations have used gamification to encourage targets of influence to complete tasks that may be viewed as boring, unpleasant, or unnecessary by making the tasks seem more rewarding and fun. For example, gamification has been used for facilitating workplace training [31] and cultivating consumer engagement [32]. “Serious games” have been receiving attention particularly in the domain of health [33,34], where the games have been used for a variety of interventions, including increasing physical activity [35,36], slowing cognitive decline [37], improving driving skills [38], and educating on self-management of health conditions [39].

Common game design features include a mechanism for providing feedback on progress toward goals, as well as rewards for achieving desired behaviors. Feedback can be in the form of scorecards that provide information on user performance, much like a performance summary page that one might encounter in a video game. It is also common for websites to encourage completion of tasks (eg, constructing user profiles) by adding a progress bar that depicts how close a user is to task completion. In a consumer behavior study, Cheema and Bagchi [40] found that the easy-to-visualize format of a progress bar may increase motivation relative to more difficult-to-visualize formats for people who are close to the goal. In addition to features that provide performance feedback and encourage task completion, another hallmark of game design is the use of playful elements that aim to elicit the experience of fun [41] and can include visuals that are amusing, interesting, provocative, or distinctive in ways that would be viewed as a pleasant surprise. Although some scholars have drawn a distinction between gamefulness and playfulness [10], in practice, users may not distinguish between them.

Because gamification encompasses multiple components in an integrated system [42], it should be noted that the focus of this pretest was not on testing an integrated gamified system designed to change the behaviors monitored by CHART. Rather, given our formative research goal, the study focused on pretesting visual elements (including some with progress bars and visually interesting elements) as potential improvements to the personalized feedback provided by CHART. In the gamification community, the term game-inspired design describes the general use of aesthetic features, narrative tones, and other elements borrowed from games in the design of other objects [43]. Therefore, we describe the infographics with these features as being game inspired rather than as part of a gamified system for this pretest.

There are compelling reasons to consider game-inspired design in the development of visuals for health behavior assessments. Game-inspired infographics can include playful elements that may elicit positive attitudes and affect. Based on the hierarchy of effects model [25], these attitudes may serve as antecedents to the desired behavior. Furthermore, game-inspired infographics may cue interpretation in ways that are helpful to health goals. The progress bar, for instance, implies the presence of a desirable goal, which is a concept that is not built into more conventional displays of quantity such as the standard column chart. In emphasizing the presence of a desirable goal, a progress bar may serve as a way to frame the advocated behavior in terms of gains from meeting the goal rather than losses from not meeting the goal. From a health perspective, the literature on gain-versus-loss framing shows that gain framing is especially well suited to prevention contexts [44], in line with the behavior change aims of the CHART intervention program. Moreover, because health behaviors are often associated with multiple and sometimes competing attitudes [45], infographics could increase the accessibility of associations that support health goals, which would in turn have the potential to strengthen the link between attitude and behavior [46].

In summary, visuals in health contexts can affect both information processing and attitudinal outcomes, and it is important to examine effects in both domains for the purposes of pretesting. Based on the studies reviewed, our formative test was guided by the following expectations and questions. First, we proposed that infographics would facilitate information processing (including comprehension, recall, and perceptions of processing ease) over text-based formats. Second, we expected that game-inspired infographics would increase engagement (attitudes toward the information, emotional tone, and perceived effectiveness) more effectively than traditional infographic formats within the context of CHART personalized health feedback. Third, to gain further insight, we also explored whether any effects of format might depend on whether an individual has already successfully met the goal for the behavior. Individuals who have not met behavioral goals are of particular importance because they represent those who might benefit the most from persuasive efforts.

## Methods

### Participants and Procedure

To test the effects of infographic format, we conducted posttest-only, between-participants experiments administered online by Qualtrics (Provo, Utah). We recruited participants (N *=* 1162) on Amazon.com’s Mechanical Turk (Amazon.com, Inc, Seattle, WA, USA), a crowdsourcing Internet marketplace for recruiting workers to complete tasks. Research has demonstrated the value of Mechanical Turk as a recruiting tool in experimental [47] and public health research [48]. The study was advertised as a short survey that offered US $1.25, which was a typical incentive for surveys of this duration at the time of data collection. Individuals were eligible if they were 18–65 years old, able to communicate in English, and living in the United States.

There were 6 concurrent experiments, with 1 for each behavior (ie, tobacco use, alcohol use, vegetable consumption, fruit consumption, physical activity, and weight management). We first randomly assigned participants to behavioral modules. Then, within modules, we randomly assigned participants to condition (traditional or game-inspired format, as described below under the Stimuli subheading). Participants answered questions assessing information processing and engagement. These were followed by items that were not related to the study and served as distractors to enable measurement of recall at the end. Finally, participants viewed items measuring potential moderators, demographics, and recall. Participants received a unique code to enter in Mechanical Turk to confirm completion. The study was approved by the University of North Carolina at Chapel Hill Institutional Review Board, where the research was conducted.

### Stimuli

#### Overall Approach

**Figure 1:**
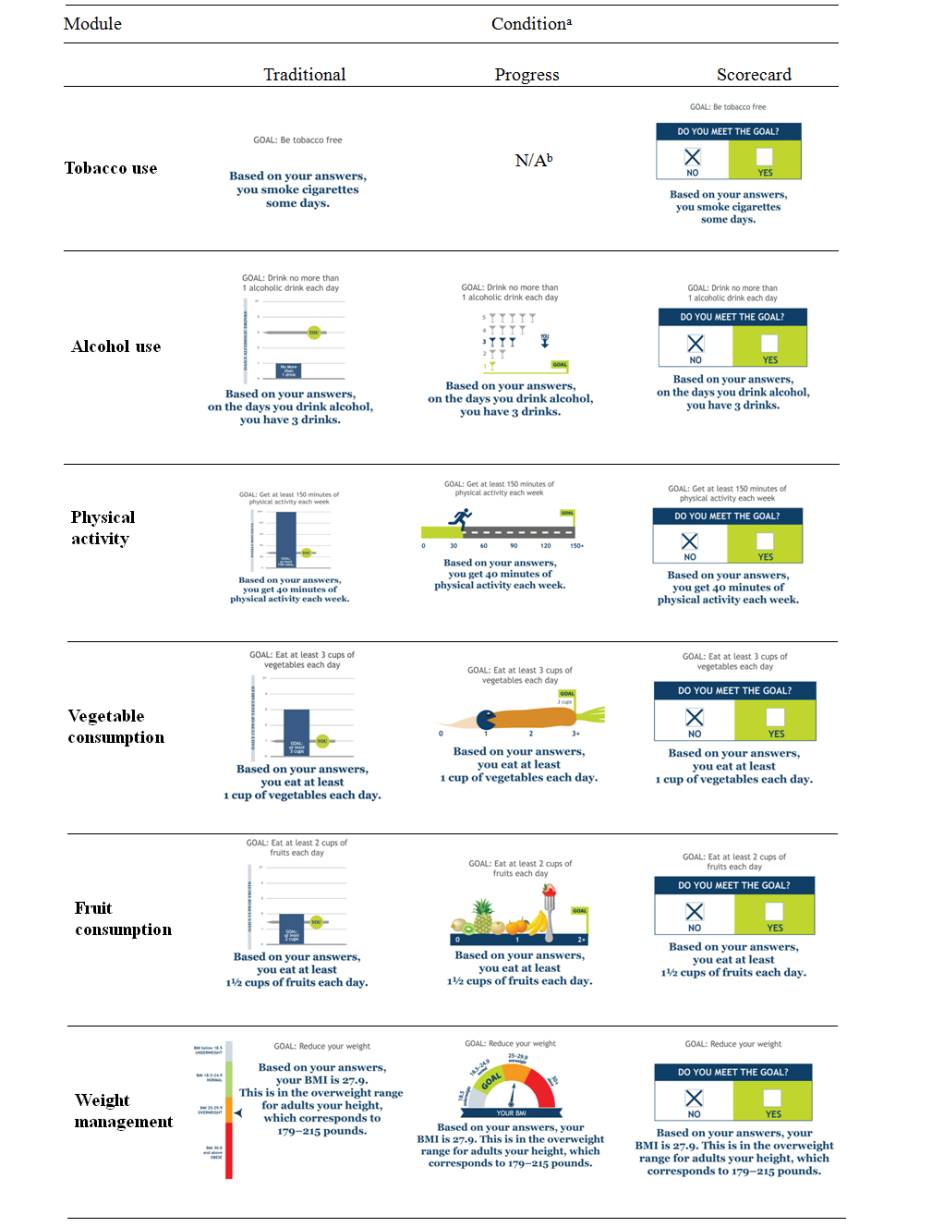
Stimuli by module and condition.^a^Conditions are traditional (text-only for tobacco; column for nontobacco), progress (game‐inspired bar), and scorecard (game‐inspired side‐by‐side comparison).^b^Progress bar format was not applicable (N/A) to tobacco module, which provides dichotomous feedback.

Stimuli were developed by the same professional graphic designer to ensure equivalence in execution quality and style. Participants were exposed to sample results of a health behavior assessment that a hypothetical individual would receive. Within each module, all of the formats presented the same level of individual behavior and the same evidence-based goal. In all modules, the hypothetical user’s behavior is short of the goal but would likely not be considered obviously unhealthy. To increase the likelihood that participants would attend to hypothetical feedback, we asked participants to imagine that the results belonged to a friend. By having the results attached to a specific person, we aimed to make the information more concrete, which is perceived as more useful and relevant to decision making than abstract information [49]. Figure 1 displays all formats used by condition and module.

#### Traditional Formats

In all modules, there was a condition that represented the existing traditional formats that were in use by CHART at the time of the study. In the tobacco module, this format was a text-only display. In the other modules, the format was a traditional column chart, with the vertical bar displaying the individual’s level of behavior and a line indicating the recommended level. The traditional column charts also included text identifying the goal and the individual’s level of behavior.

#### Game-Inspired Formats

To serve as comparisons with traditional formats, we created 2 game-inspired formats. In the *scorecard* format, the game-inspired element was the visual appearance of a game scorecard. As adapted for this study, the scorecard included a side-by-side display of “yes” and “no” checkboxes to indicate whether a person has met the goal. This visual was accompanied by text that stated the goal and the individual’s level of behavior. We used the scorecard format in all modules in this executional style. In the *progress bar* format, a game-inspired element was the gamification concept of a horizontal bar displaying the level of achievement toward task completion. As with the scorecard, this format also included text that stated the goal and the individual’s level. We did not use the progress bar format in the tobacco module, which provides only dichotomous yes/no feedback of whether one has met the goal of being tobacco free. However, we did use the progress bar in all other modules, which provide feedback on an ordinal numerical scale.

In addition to the visual appearance of a progress bar, other game-inspired features in this format included playful elements and cues to action that were consistent with the advocated behaviors. For example, the progress bar for eating fruits included pieces of fruit and a fork, and the progress bar for physical activity was represented by a figure running toward a finish line. Although the general concept of a progress bar entails an increase from left to right toward a fixed goal, we had to modify the concept for the modules of weight and alcohol because, in these cases, behavioral goals do not involve a straightforward increase. Rather, alcohol goals require ensuring that use does not exceed a particular level, while weight goals require keeping body mass index within a healthy range. Thus, modifications of the progress bar were developed for greater congruence with these goals.

Game-inspired conditions in each module were represented by single exemplars, which we chose as follows. The graphic designer developed several mock-ups as candidates to represent each condition. For the progress bar executions, the designer was instructed to include playful elements and behavioral cues where appropriate. The research team reviewed all options and selected the one that was the clearest and most reasonable example of each condition, given the behavior and the overall context of the website. The research team suggested modifications to the designs and reviewed final versions prior to use.

### Dependent Measures

#### Information Processing

*Comprehension* was measured with multiple-choice questions that appeared along with the infographic. The purpose was to assess whether participants could interpret the graphic correctly. Questions asked what the health goal is, whether the goal was met, and (if appropriate for the module) whether meeting the goal required an increase or decrease in current behavior and by how much. The number of correct answers was summed. Because there was a large proportion of all-correct responses (reported in results), we dichotomized the variable as all-correct or not.

*Recall* of the goal was measured at the end of the survey with a single item that was not accompanied by the feedback display viewed earlier. The item appeared after many distractor items, and the purpose was to assess whether information could be retrieved. We coded responses as correct or not.

*Time to answer comprehension questions* was collected as an unobtrusive measure of information processing ease, with shorter times indicating greater ease. Collection of this data was made possible by selecting the option in Qualtrics that records response times to questions. Values were adjusted for the number of questions in the module. Also, because response latencies are typically skewed, we log transformed the data before analysis, per standard practice [50].

*Perceived cognitive load* measures self-reported difficulty in processing the information [51]. The following items appeared immediately after the comprehension questions: (1) “I had difficulty understanding the infographic,” (2) “I felt ‘lost’ when interpreting the infographic,” and (3) “It was clear how the information fit together” (reverse coded). Response options ranged from 1 (not at all) to 5 (a lot). Items were averaged (Cronbach alpha = .82).

#### Engagement

*Attitude toward the infographic* was measured with 7 bipolar adjectives adapted from the Attitude Toward the Ad scale [30]. Participants were asked to what extent (range 1–5) they thought the infographic looked not cool/cool, boring/interesting, unpleasant/pleasant, unappealing/appealing, not likable/likable, unexciting/exciting, and unattractive/attractive. Items were averaged (Cronbach alpha = .94).

*Positive emotional tone* included 3 items adapted from Pechmann and Reibling [52]. Participants were asked to what extent looking at the infographic made them feel amused, happy, and upbeat. Response options ranged from 1 (not at all) to 5 (a lot). Items were averaged (Cronbach alpha = .88).

*Perceived effectiveness* was measured with 3 items that asked the extent to which participants thought the infographic would be an effective way to provide information, would be valuable to the recipient, and would be motivating. The items are similar to those used in assessments of persuasive messages [53]. Response options ranged from 1 (not at all) to 5 (a lot). Items were averaged (Cronbach alpha = .88).

### Moderator Variable

*Participants’ own behavior* was assessed in the module to which they were assigned with the same items used in CHART to assess that behavior. We adapted these items from validated sources and describe them here briefly. For tobacco, we asked participants whether they smoked cigarettes every day, some days, or not at all; current smoking was defined as using every day or some days [54]. For alcohol, we asked participants about consumption in the past 30 days [54]. For physical activity, participants provided the number of minutes of physical activity they get in a typical week [55]. For the modules of vegetable and fruit consumption, participants reported how many cups they eat in a typical day [56]. For weight management, participants reported height and weight, which we used to compute their body-mass index. For all modules, we coded responses as meeting the goal or not. The goals for each behavior appear on the infographics in Figure 1. Percentages of participants not meeting goals are reported below.

### Analysis Plan

#### General Approach

Although the 6 between-participants experiments conducted in this pretest would essentially allow analysis of multiple replication studies, such an approach would be lengthy to report and would not answer the question of which format overall would be best suited to the website and whether there were boundary conditions imposed by module on condition effects. Thus, we proceeded as follows. We analyzed the data in the tobacco and nontobacco modules separately because they differed in number of conditions compared (2 in tobacco, 3 in nontobacco). For data in nontobacco modules, we first examined the interaction of module and condition to see whether the effect of condition depended on module. There was no significant interaction, indicating the consistency of effects across the experiments in the nontobacco modules; therefore, we combined the data. A fixed-effects approach was used given the small number of nonrandom messages employed (see [57], for arguments supporting fixed-effects over random-effects approaches in similar contexts). We included module as a covariate to account for possible differences in outcomes based solely on module. There were no differences in distributions of demographic variables across the study groups (*P* values ranging from .15 to .80). As a result, we did not use demographics and other attributes as covariates.

#### Main Effects of Condition

For categorical outcome variables of comprehension and recall, we used logistic regression to model the effect of infographic condition on each outcome. For the continuous information processing outcomes (cognitive load and time to complete), we first ran multivariate analysis of variance to evaluate the overall effect of condition. If the Wilks’ lambda test was significant, we ran univariate analysis of variance to evaluate the effect on individual outcomes. Significant univariate results were followed by pairwise comparisons when there were more than 2 groups being compared (ie, for nontobacco data). We used the same process for the continuous outcomes related to engagement (attitude, positive emotional tone, and perceived effectiveness).

#### Moderation of Condition Effects

We evaluated the potential moderator of whether one has met the goal by using an interaction term. If we found a significant interaction, we report results for the effect of infographic separately for those who did and did not meet the goal.

## Results

### Sample Characteristics and Assignment to Module/Condition

Table 1 summarizes sample characteristics. Table 2 provides the percentages of participants who did not meet the behavioral goals for each module. The percentages range from 25% for tobacco (ie, 25% used tobacco) to 86% for vegetable consumption (ie, 86% did not eat the recommended amount). Table 2 also shows the numbers of participants who were randomly assigned to each module and to each condition within the module.

### Main Effects of Condition

#### Tobacco

**Figure 2:**
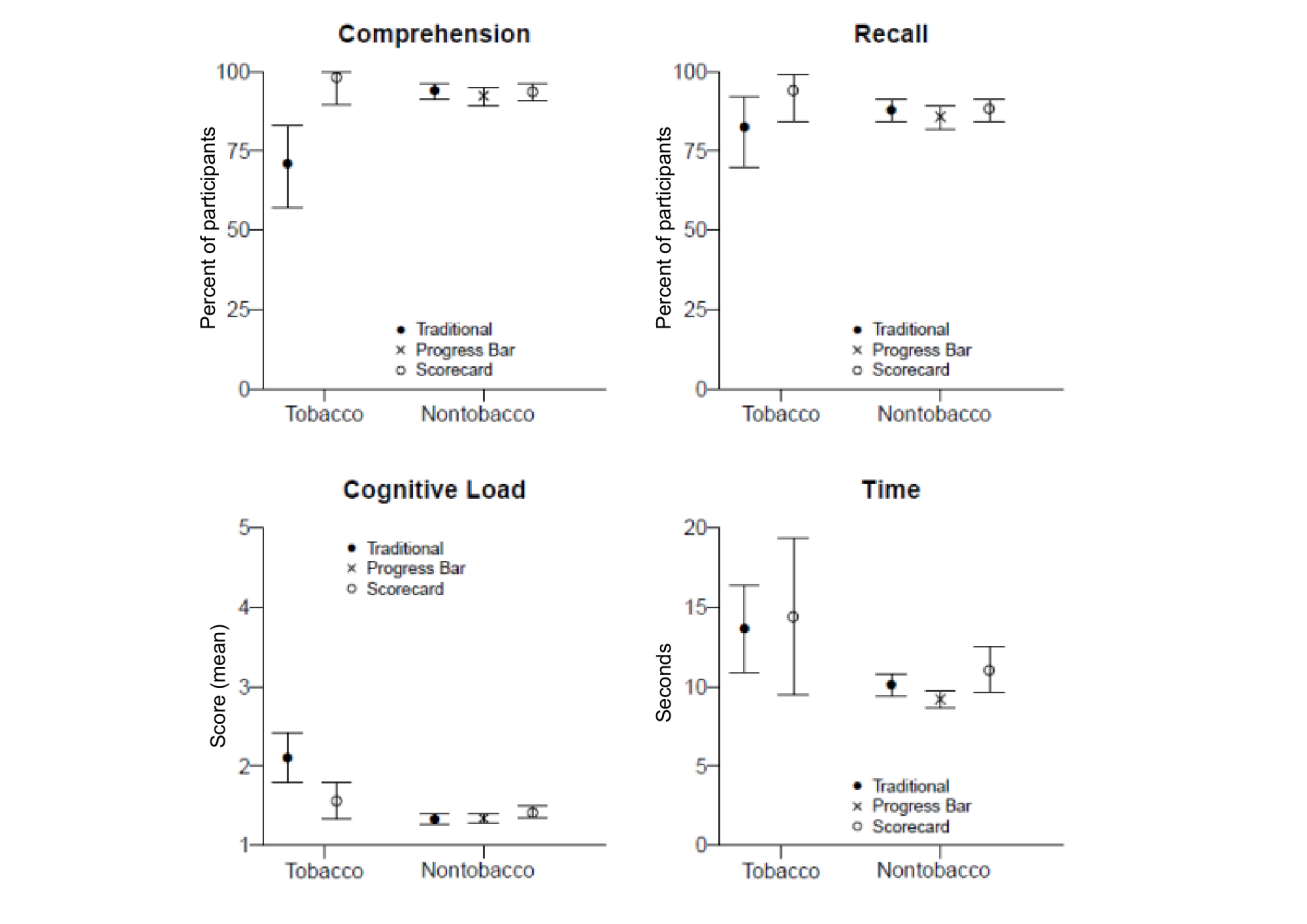
Percentages and means for information processing outcomes by condition and module. Bars extend to upper and lower bounds of the 95% CIs. For comprehension and recall, percentages reflect those who answered correctly. For perceived cognitive load, means are on a 1–5 scale; lower means denote lower perceived load (ie, easier processing). Time to answer comprehension questions was measured in seconds and was adjusted for number of questions in the module; lower means denote less time taken to answer (ie, easier processing). Traditional condition was text-only for the tobacco module, and traditional column chart for nontobacco modules.

We compared 2 conditions: game-inspired (scorecard) and traditional (text-only). For information processing outcomes, participants who viewed the scorecard (vs text-only) were more likely to get all comprehension questions correct (98.1% vs 71.2%, *P=*.004) and to get the recall question correct (94.2% vs 82.7%), although the difference in percentages for recall was only marginally significant (*P*=.08). Multivariate analysis showed a significant association of infographic type on the outcomes of perceived cognitive load and time to answer questions (*P*=.02). Univariate analyses revealed that participants who viewed the scorecard reported lower cognitive load than did those who viewed text-only (estimate –.54, *P*=.006). However, in terms of time to answer, the difference was not significant (*P*=.70). Figure 2 shows comparisons of means and percentages for information processing outcomes by condition.

**Figure 3:**
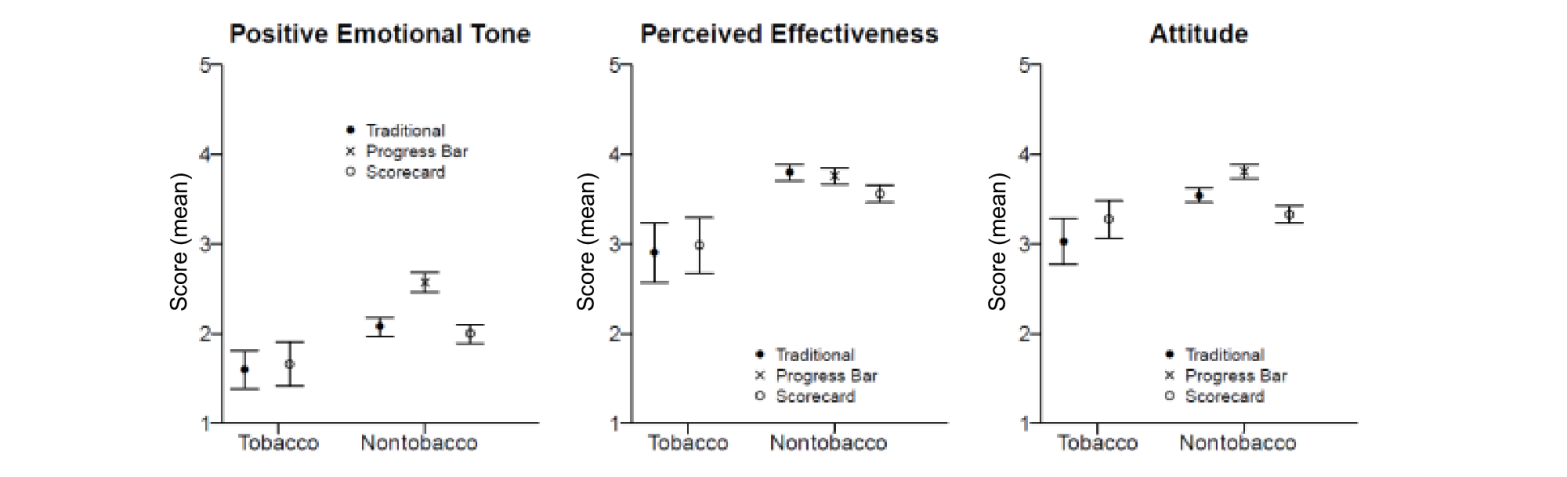
Means for engagement outcomes by condition and module. Bars extend to upper and lower bounds of the 95% CIs. Higher means denote more positive emotional tone, greater perceived effectiveness, and more positive attitude toward the infographic. Traditional condition was text-only for the tobacco module and traditional column chart for nontobacco modules.

For engagement-related outcomes, multivariate analysis with the 3 dependent variables (attitude toward the infographic, positive emotional tone, and perceived effectiveness) did not reveal an effect of condition (*P*=.42), so we did not probe them further (Figure 3).

**Table 1:** Characteristics of participants (N=1162) in experiments assessing infographics.

Characteristic		Mean (SD)	No.	%
Age in years		32.5 (11.1)		
**Sex**				
	Male		624	53.70
	Female		538	46.30
**Race**				
	White		939	80.81
	Black		89	7.66
	American Indian		5	0.43
	Asian		84	7.23
	Native American		7	0.60
	Hawaiian/Pacific Islander		6	0.52
	Multiple		32	2.75
**Ethnicity**				
	Hispanic		76	6.54
	Non-Hispanic		1085	93.37
**Education**				
	Less than high school		6	0.52
	High school/GED^a^		115	9.90
	Some college		341	29.35
	2-year college		127	10.93
	4-year college		452	38.90
	Master’s degree		92	7.92
	PhD, JD, or MD degree		29	2.50

^a^General equivalency diploma.

**Table 2:** Cell sizes by module and condition, and percentages of participants not meeting behavioral goals by module.

Module	Condition^a^			No. (%)^b^	No. (%) not meeting goal
	Traditional	Progress	Scorecard		
Tobacco use	52	N/A^c^	52	104 (8.95)	26 (25.0)
Alcohol use	58	59	58	175 (15.06)	88 (50.3)
Physical activity	56	60	55	171 (14.72)	93 (54.4)
Vegetable consumption	51	52	53	156 (13.43)	134 (85.9)
Fruit consumption^d^	96	88	93	277 (23.84)	195 (70.4)
Weight management^d^	95	91	93	279 (24.01)	154 (55.2)

^a^Conditions are traditional (text-only for tobacco; column for nontobacco), progress (game‐inspired bar), and scorecard (game‐inspired side‐by‐side comparison).

^b^Total participants for all modules: N=1162.

^c^Progress bar format was not applicable (N/A) to tobacco module, which provides dichotomous feedback.

^d^More participants were recruited for modules of fruits and weight (relative to other modules) to allow for analysis of variables not related to this study.

#### Health Behaviors Other Than Tobacco

We compared 3 conditions: 2 game-inspired (scorecard and progress bar) and 1 traditional (column chart). For information processing outcomes, both comprehension and recall (≥85%) were high across conditions, and pairwise contrasts among the 3 conditions did not show differences (all *P* ≥.20 for both outcomes). Multivariate analysis of perceived cognitive load and time to answer questions was not significant (*P*=.09) (Figure 2).

For engagement-related outcomes, multivariate analysis revealed a significant association (*P*<.001), and univariate analyses revealed significant differences for each of the 3 outcomes (attitude toward the infographic, positive emotional tone, and perceived effectiveness) (Figure 3).

For attitude toward the infographic, participants who viewed the progress bar had the most positive attitudes, followed by those who saw the traditional bar, and then the scorecard (mean scores 3.81, 3.54, and 3.33, respectively). Pairwise contrasts showed that all means were significantly different from each other (*P*<.001 for all).

For positive emotional tone, participants who viewed the progress bar gave the highest ratings (ie, extent to which the infographic made them feel amused, happy, and upbeat), followed by those who saw the traditional bar, and then the scorecard (mean scores 2.57, 2.08, and 2.00, respectively). The significant contrasts were progress versus scorecard, and progress versus traditional bar (*P*<.001 for both contrasts).

For perceived effectiveness, there were no differences between participants who viewed the progress bar and the traditional bar (in terms of perceptions of providing information, motivating behavior, and being valuable). The mean scores were 3.76 and 3.80 (*P=*.59). The scorecard had the lowest mean on this outcome (mean 3.56), and it was significantly lower than the means for progress and traditional bar (*P*=.004 and *P*<.001, respectively).

### Moderation of Condition Effects

Finally, we asked whether any effects of infographic condition would be moderated by whether the individual has met the behavioral goal. No effect was seen for having met the goal in the tobacco module, but for the nontobacco modules, having met the goal influenced the relationship between infographic condition and positive emotional tone (interaction *P=*.003). Because the interaction indicates that the effect of condition changes as a function of whether the goal has been met, we probed the interaction to understand the nature of the difference.

Results showed that, regardless of whether or not one had met the goal, the progress bar performed best; however, differences between the progress bar and the other 2 formats were more pronounced for those who did *not* meet the goal. The mean scores for the progress bar, traditional bar, and scorecard were 2.69, 1.98, and 1.96, respectively, for those who did not meet the goal, and 2.38, 2.24, and 2.08, respectively, for those who did meet the goal. The progress bar was rated significantly higher than both the traditional bar and the scorecard among those who did not meet the goal (both *P*<.001), and significantly higher than the scorecard among those who did meet the goal (*P=*.01).

## Discussion

Health behavior assessments that provide personalized feedback face the challenge of conveying to individuals how their behaviors stack up to evidence-based recommendations and motivating behavior change. We conducted this study as formative research to examine whether game-inspired infographics would be more or less effective than traditional formats in their ability to convey information in ways that are easy to process and engaging. We report these results to document the process of development and to potentially benefit other eHealth programs that seek to convey similar information in similar contexts.

We found that, across multiple health behaviors, a game-inspired infographic was superior to a traditional format for some outcomes, and for other outcomes there was no observable difference. Specifically, in the tobacco module, where the scorecard was the only game-inspired design that we tested, the scorecard outperformed the traditional text-only format on comprehension and perceived cognitive load, and there were no differences in performance on the other information processing and engagement outcomes. In the modules for behaviors other than tobacco, there were no differences between the progress bar and the traditional bar for the information processing outcomes, but the progress bar outperformed the traditional bar on outcomes related to engagement (positive emotional tone and attitude). Thus, based on the potential for a game-inspired design to outperform in the cases described above (and not to underperform otherwise), game-inspired designs show promise as a component of behavioral assessment output, relative to more traditional formats.

Moreover, in the nontobacco modules, the appeal of the progress bar over the traditional was pronounced among people who had not yet met the national recommendation for that health behavior. Individuals in this group may represent a priority audience because they would benefit the most from persuasive efforts. Prior work suggests that intervention efforts aimed at people who see no need to change health behavior should emphasize positive consequences of change more than negative consequences of not changing [58]. Game-inspired infographics that explicitly frame feedback in a positive light (eg, progress toward a desirable state) may therefore be more effective with this group than other formats, consistent with our results.

Comparing the findings for tobacco and nontobacco modules, a question that arises is why there were effects on information processing but not engagement outcomes in the tobacco module, and on engagement but not information processing outcomes in the nontobacco modules. The asymmetry may be due to the comparisons that were available in the tobacco versus nontobacco modules. In terms of information processing, the comparisons in the nontobacco modules were all among equally information-rich conditions, in the sense that all conditions provided 3 informational components (goal statement, whether the goal was met, and a visual representation of that information). In contrast, the comparison available in the tobacco module was between 2 conditions that differed in information richness, in the sense that the text-only traditional condition (which provided only 2 components: the goal statement and whether the goal was met) was compared with text plus visual (3 components) in the game-inspired scorecard condition. Thus, it makes sense that we observed a difference in information processing in the tobacco module but not in the other modules, as would be expected based on dual coding theory [21].

Similarly, in terms of engagement outcomes, both conditions in the tobacco module lacked design elements that may have contributed to emotional appeal, whereas the progress bar conditions in the nontobacco modules were designed to contain such elements (eg, playful design and sense of movement toward a desirable goal). Therefore, it makes sense that we observed differences on this dimension in nontobacco modules but not in the tobacco module. The dichotomous feedback in the tobacco module ruled out a progress bar as a potential format in this study, so we used only the scorecard. However, future research could examine the effectiveness of other visual representations of tobacco cessation goal attainment that may convey greater emotional appeal. In the nontobacco modules, it is interesting that the scorecard fared worse than the traditional bar on 2 of the 3 engagement outcomes. It may be that the dichotomization of behavior in the scorecard is less appealing than formats that present feedback within a numerical range, as both other formats did. However, it is clear that the progress bar outperformed the scorecard on all 3 engagement outcomes and is therefore the more promising of the 2 game-inspired formats in the context of this study. The differences in performance between these 2 game-inspired formats further highlight the value of pretesting different executions during the formative research process.

### Limitations

It is important to address the limitations introduced by the exploratory nature of the study. We used Mechanical Turk as a recruitment tool for this initial test because Mechanical Turk made it possible to obtain large samples at a reasonable cost; however, the resulting sample was not as diverse as would have been ideal in terms of age, race, ethnicity, and education level. Future research should employ methods that would yield a more diverse sample. The design of the study was posttest-only, which does not allow observation of changes before and after exposure. Although a posttest-only design helps avoid some potential threats to validity that might be introduced by preexposure measures (such as sensitizing individuals to treatment or later measurement), future studies should take a longitudinal approach to enable the observation of changes in key variables over time.

In terms of stimuli, we used only one execution to represent each format in each module, and although we selected the game-inspired exemplars as the best representatives of the formats among many alternatives reviewed by the research team, it is possible that other executions would have been more effective. We combined modules in the analysis after ensuring that the effect of condition did not differ across modules; as a consequence, though, it is not possible to pinpoint which specific features of the game-inspired infographics were responsible for observed effects. Subsequent research should systematically test multiple versions in which elements are present or absent to identify which had the most impact. Participants viewed sample feedback that was standardized within a module for experimental control and not tailored to their own behavior because it was not feasible to produce all possible variations across modules, formats, and behavior levels. However, we are planning research to evaluate the infographics on the actual CHART platform, which would provide personalized assessments and would also enable us to study the effects of infographics on intentions and outcomes related to behavior change.

### Conclusions

The personalized feedback provided by health behavior assessments must tell people who do not meet a behavioral goal that they are short of the mark without leading to demotivation and disengagement. This is a challenging task. The principles of game design informed the development of infographics that were better than or did not differ from traditional formats in terms of effects on engagement and information processing outcomes. The results of our pretest have the potential to help online health assessment tools provide personalized feedback in a way that may facilitate progress toward health goals.

## Abbreviations

CHART: Carolina Health Assessment and Resource Tool
