# Acknowledgment Correction of: Impact of Game-Inspired Infographics on User Engagement and Information Processing in an eHealth Program

**DOI:** 10.2196/jmir.7104

**Published:** 2017-01-09

**Authors:** Maria Leonora G Comello, Xiaokun Qian, Allison M Deal, Kurt M Ribisl, Laura A Linnan, Deborah F Tate

**Affiliations:** ^1^ School of Media and Journalism University of North Carolina at Chapel Hill Chapel Hill, NC United States; ^2^ Lineberger Comprehensive Cancer Center University of North Carolina at Chapel Hill Chapel Hill, NC United States; ^3^ Gillings School of Global Public Health University of North Carolina at Chapel Hill Chapel Hill, NC United States

The authors of “Impact of Game-Inspired Infographics on User Engagement and Information Processing in an eHealth Program” (J Med Internet Res 2016;18(9):e237) would like to change the acknowledgments section of their paper to the following: 

“This research was supported by the University Cancer Research Fund. For graphic design and software development, the authors thank UNC CHAI Core, which is supported in part by a grant from NIH (DK056350) to the University of North Carolina Nutrition Obesity Research Center and from NCI (P30-CA16086) to the Lineberger Comprehensive Cancer Center. The authors are grateful to Barbara Alvarez Martin, MPH, and Anne Cabell, MPH, for their assistance with the study. The first author also thanks Deanna Puglia for assistance preparing the manuscript.”

This correction has been made in the online version of the paper on the JMIR website on January 09, 2017, together with publishing this corrigendum.

A correction notice has been sent to PubMed, and the publication was resubmitted to PubMed Central and other full-text repositories.

